# Impact of maternal HIV-1 viremia on lymphocyte subsets among HIV-exposed uninfected infants: protective mechanism or immunodeficiency

**DOI:** 10.1186/1471-2334-14-236

**Published:** 2014-05-05

**Authors:** Fatima Kakkar, Valerie Lamarre, Thierry Ducruet, Marc Boucher, Silvie Valois, Hugo Soudeyns, Normand Lapointe

**Affiliations:** 1 Division of Infectious Diseases, CHU Sainte-Justine, 3175 Côte Sainte-Catherine, Montreal, Quebec H3T 1C5, Canada; 2 Department of Pediatrics, Faculty of Medicine, Université de Montréal, Montreal, Canada; 3 Centre maternel et infantile sur le SIDA, CHU Sainte-Justine, Montreal, Canada; 4 Unité de recherche clinique appliquée, CHU Sainte-Justine, Montreal, Canada; 5 Division of Obstetrics and Gynecology, CHU Sainte-Justine, Montreal, Canada; 6 Unité d’immunopathologie virale, Centre de recherche du CHU Sainte-Justine, Montreal, Canada; 7 Department of Microbiology, Infectious Diseases and Immunology, Faculty of Medicine, Université de Montréal, Montreal, Canada

**Keywords:** Mother-to-child HIV human immunodeficiency virus transmission, Antiretroviral drugs, Viral load, Immune suppression, Immune system

## Abstract

**Background:**

Reports of increased morbidity and mortality from infectious diseases among HIV Exposed Uninfected (HEU) infants have raised concern about a possible underlying immunodeficiency among them. The objective of this study was to assess the immunological profile of HEU infants born to mothers exhibiting different levels of HIV-1 viremia at the time of delivery.

**Methods:**

Study subjects were enrolled in the Centre maternel et infantile sur le SIDA (CMIS) mother-child cohort between 1997 and 2010 (n =585). Infant CD4^+^ T cell, CD8^+^ T cell and CD19^+^ B cell counts were assessed at 2 and 6 months of age, and compared among HEU infants in groups defined by maternal viral load (VL) at the time of delivery (VL < 50 copies/ml, VL 50–1000 copies/ml, and VL > 1000 copies/ml) in a multivariable analysis.

**Results:**

At 2 months of age, infants born to mothers with VL > 1000 copies/ml had lower CD4^+^ T cell counts compared to those born to mothers with VL < 50 copies/ml at the time of delivery (44.3% *versus* 48.3%, p = 0.007, and 2884 vs. 2432 cells/mm^3^, p = 0.02). These differences remained significant after adjusting for maternal and infant antiretroviral drug use, gender, race and gestational age, and persisted at 6 months of age. There were no differences in CD8^+^ T cell count or absolute CD19+ B cell count between groups, though higher CD19+ B cell percentage was seen among infants born to mothers with VL > 1000 copies/ml.

**Conclusions:**

These results suggest that exposure to high levels of HIV-1 viremia *in utero*, even in the absence of perinatal transmission, may affect the infant’s developing immune system. While further work needs to be done to confirm these findings, they reinforce the need for optimal treatment of HIV infected pregnant women, and careful follow-up of HEU infants.

## Background

The development and successful implementation of prevention of mother-to-child transmission (PMTCT) programs has reduced the risk of perinatal transmission of HIV-1 from 25-30% to less than 1% [1,2]. In Canada, as in many parts of the developed world, HIV-exposed uninfected (HEU) infants significantly outnumber those who are infected [3]. However, despite successfully averting HIV-1 infection, there is emerging concern about the health of HEU infants [4]. Reports from South Africa, the Caribbean, South-East Asia and Europe have found increased morbidity and mortality from infectious diseases among HEU infants [5-9]. HEU infants have been found to have a higher incidence of neonatal infections, neonatal sepsis, respiratory illness, acute gastroenteritis, hospitalization, and post-operative complications compared to the general population [10-15], and compared to HIV-unexposed uninfected controls [16,17]. Perhaps most concerning are reports of pneumonia caused by *Pneumocystis jiroveci*, normally considered to be an HIV-associated opportunistic infection, among HEU infants [18-20].

While the cause of this increased morbidity and mortality from infectious diseases among HEU infants is likely to be multifactorial, encompassing environmental, maternal, and health systems factors, the increased burden of infections seen among them raises the question of whether a relative state of immunodeficiency may contribute to their increased susceptibility to disease [21]. A number of studies to date have shown evidence of various immunological abnormalities among HEU children. The most consistent findings have been markers of increased immune activation and reduced total and naive CD4^+^ T cell counts in HEU newborns [22-26]. It is hypothesized that these immunological changes may result from a combination of factors whereby the *in utero* environment of HIV infected mothers uniquely shapes their infant’s immune system. Previous studies have shown an association between maternal antiretroviral (ART) drug use and lower infant CD4 counts and thymic CD4 output [27,28], maternal CD4 count and infant CD4 count [29,30], and maternal recreational drug use and lower infant CD4 counts [31], though few have studied the impact of maternal HIV-1 viremia on infant immunological parameters. However, there is increasing evidence across a range of infectious diseases (viral, bacterial and parasitic) to suggest that chronic maternal infections during pregnancy can affect an infant’s immune response to future infections, independently of the vertical transmission of pathogens [32]. *In utero* sensitization of the fetal immune system to maternal pathogens is thought to lead to altered cytokine, proliferative, and antibody responses to these pathogens in the infant [33-35]. There is evidence that HEU infants are exposed to HIV-1 *in utero;* circulating HIV-1 proteins and/or immune complexes have been shown to induce strong HIV-specific T helper and cytotoxic T cell responses in HEU newborns, when these infants clearly are uninfected [36-39].

Given the reports of increased morbidity and mortality from infectious diseases among HEU infants, and our limited understanding on the nature and cause of immune abnormalities in these children, the objective of the present study was to test the hypothesis that is *in utero* exposure to high levels of HIV-1 viremia may drive the immunological changes seen among HEU, in the absence of acute infection.

## Methods

### Study population

The Centre maternel et infantile sur le SIDA (CMIS) Mother-Child Cohort was established in 1988 to prospectively follow all pregnant HIV-infected women and their newborns presenting to Centre hospitalier universitaire (CHU) Sainte-Justine, a tertiary referral center in Montreal, Canada. HIV infected women were treated by a mutlidisplinary obstetrics-infectious diseases team on site, and HIV-1 plasma RNA levels at the time of delivery were measured in real time using the Versant HIV-1 RNA assay (Bayer, Pittsburg), with a detection threshold of 2.70 log10 (500) HIV-1 RNA copies/ml (version 2.0, 1997–2000), or 1.70 log10 (50) HIV RNA copies/ml plasma (version 3.0, 2000–2010).

HIV exposed infants were followed closely in the first 2 years of life to determine their HIV status and to monitor for the effects of *in utero* and post-natal exposure to ART. HIV infection status in infants was determined using 4 negative HIV-1 DNA PCR tests (2 weeks, 1 month, 2 month and 4 months of life) (Roche Amplicor), and the absence of HIV-1 antibodies by ELISA at or after 18 months of age. All infants in the cohort were exclusively formula fed, and received 6 weeks of ART for prevention of mother-to-child transmission as soon as it became available. This resulted in different neonatal treatment regimens over time. From 1994–1997, infants at our center received AZT alone, from 1998–1999 AZT in combination with 3TC, and from 1999–2007, AZT, 3TC and Nelfinavir. Following the recall of Nelfinavir in 2007, the neonatal regimen changed again from 2007–2010 to a combination of AZT and 3TC. Clinical and laboratory monitoring included complete physical exam at every visit, routine haematology and biochemistry tests until 18 months of age, and numeration of lymphocyte subsets (CD4^+^ T cells, CD8^+^ T cells, CD19^+^ B cells) by flow cytometry at 2 and 6 months of age. After 2 years of age, children were offered annual pediatric follow up until age 5, and every 2 years thereafter. Demographic and laboratory data were collected at every visit and entered into the CMIS database. Informed consent was sought and obtained from all participants to the CMIS Cohort. This protocol was reviewed and approved by the Ethics Review Board of CHU Sainte-Justine, Montreal, Canada, where the study was conducted.

**Figure 1:**
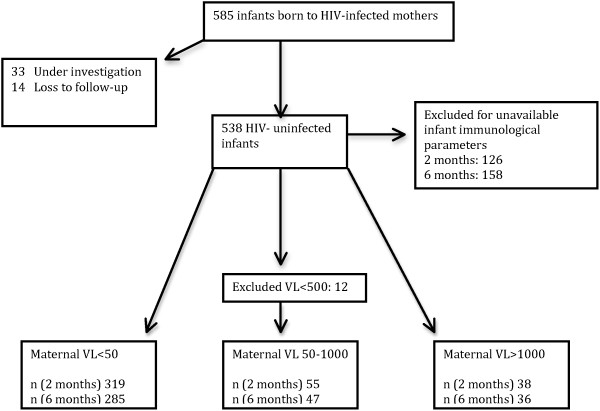
Participant enrollment and flow diagram for mother-infant pairs included in the analysis at 2 and 6 months of age.

All HEU infants born between 1997 and 2010 were eligible for the study, though infants for whom immunological data was not available at the specified ages of 2 and 6 months (due to visits outside of the routine schedule) were excluded from the analysis, given the large variability in infant lymphocyte subsets over the first few months of life. Out of a total 585 mother-infant pairs, 424 mother-infant pairs were assessed at 2 months of age and 380 at 6 months of age (Figure 1).

### Statistical analysis

Descriptive statistics were used to characterize the study population. For continuous variables, means and ranges were reported. For discrete variables, percentages were reported. For the primary analysis, infants were then categorized into 3 groups based on maternal VL closest to delivery, *i.e.* > 1,000, 50–1,000, and < 50 HIV-1 RNA copies/ml plasma. Given the changes in the sensitivity of VL testing over time, infants born to mothers with VL <500 HIV-1 RNA copies/ml plasma (n = 12), while considered undetectable at the time, were removed from the analysis given that we could not be certain about their true VL, and in order to ensure heterogeneity within strata. Infant CD4^+^ T cell, CD8^+^ T cell, and CD19^+^ B cell counts (relative and absolute) were then compared among infants in each of these 3 maternal VL strata using one-way analysis of variance. Multivariable analysis of variance was conducted to adjust for confounders identified *a priori* from published literature [22-26], including maternal CD4^+^ T cell counts, exposure to ART (mother and infant), gestational age, gender and race. The different models used in the final analysis and the significance of each variable included are presented as supplementary information in an Additional file 1: Table S1. All statistical tests were 2-sided and statistical significance threshold was set at p < 0.05. All data were analyzed using SAS version 9.2 (SAS Institute, Cary, NC).

## Results

### Maternal characteristics

Among HIV-infected mothers, 12.3% received no ART, 13.2% were treated with zidovudine (AZT) monotherapy, 8.4% with double combination therapy (AZT and lamivudine [3TC]), and 66.2% received combination ART (cART) during pregnancy. Median maternal age was 31 years (IQ = 27–34 years). 71.9% of mothers were categorized as not immunosuppressed at delivery (CD4 > 350 cells/mm^3^), while 19.7% were moderately immunosuppressed (CD4 = 200–350 cells/mm^3^) and 8.4% were severely immunosuppressed (CD4 < 200 cells/mm^3^). HIV-1 VL at delivery was undetectable (<50 HIV-1 RNA copies/ml plasma) in 77.7% of mothers, 11.7% had a VL between 50 and 1,000 HIV-1 copies/ml plasma, and 10.4% had VL > 1,000 HIV-1 RNA copies/ml plasma. Among those with VL >1000 copies/ml, median VL was 6352 (IQR 2600–10433 copies/ml). Maternal characteristics according to delivery viral load are summarized in Table 1.

**Table 1:** Maternal characteristics according to delivery viral load

**Maternal group**	**Viral load <50 copies/ml**	**Viral load 50–1000 copies/ml**	**Viral load >1000 copies/ml**
**Median viral load (IQR)**	NA	244 (105–506)	6 252 (2600 – 10433)
**Maternal age**			
(Mean, SD)	31.5 (5.5)	29.8 (5.3)	30.8 (5.2)
**Delivery CD4 count (n, %)**			
>350	276 (80.5)	34 (63.0)	27 (58.7)
200-350	49 (14.3)	9 (16.7)	8 (17.4)
<200	18 (2.2)	11 (20.4)	11 (23.9)
**Gestational age**			
(Mean, SD)	38.5 (2.2)	38.1 (1.9)	37.7 (2.7)
**Pre-term (n, %)**			
(<37 weeks)	50 (14.1)	9 (16.4)	9 (18.8)
**ART regimen**			
None	3 (0.9)	2 (3.7)	6 (12.5)
AZT	5 (1.4)	3 (5.6)	7 (14.6)
AZT/3TC	27 (7.6)	6 (11.1)	4 (8.3)
cART	319 (90.1)	43 (79.6)	21 (64.6)

### Infant characteristics

46.7% of infants were female and 53.3% were male. 29.7% were identified as Caucasian, 63.2% were black, 4.6% were mixed, and 2.5% were classified as other. When grouped by region of origin, 33.1% of infants were classified as African, 31.5% were Haitian, 28.4% Canadian, and the remaining 7.0% mixed-Canadian or other. With respect to ART given to newborns for PMTCT, 12.9% received no ART, 11.5% received AZT monotherapy, 28.7% received AZT and 3TC, and 46.9% received 3 or more antiretroviral agents.

### Lymphocyte subsets

#### Infant CD4^+^ T cell counts

There was a significant difference in CD4^+^ T cell counts at both 2 and 6 months of age among infants born to mothers with HIV-1 VL > 1,000 *vs.* < 50 HIV-1 RNA copies/ml plasma (Tables 2 and 3). At 2 months of age, infants born to mothers with undetectable VL had higher CD4 counts compared to those with VL > 1,000 HIV-1 RNA copies/ml plasma (48.3% vs. 44.3%, p = 0.007 and 2884 vs. 2432 cells/mm^3^, p = 0.02). These differences persisted at 6 months of age (46.1% *vs.* 42.1%, p = 0.009, and 2887 vs. 2271 cells/mm^3^, p = 0.002), and remained statistically significant after adjusting for gestational age, race, gender, maternal and infant ART exposure, and maternal CD4^+^ T cell counts at the time of delivery.

**Table 2:** Infant CD4, CD19 and CD8 parameters at 2 months of age

**Maternal viral load**		**CD4 (mean, 95% CI)**	**p**^**†**^	**Adjusted CD4 (mean, 95% CI)**	**p**^**‡**^	**CD19 (mean, 95% CI)**	**p**^**†**^	**Adjusted CD19 (mean, 95% CI)**	**p**^**‡**^	**CD8 (mean, 95% CI)**	**p**^**†**^	**Adjusted CD8 (mean, 95% CI)**	**P**^**‡**^
**>1000**	Relative*	44.3 (41.5–47.0)	0.007	43.5 (40.6–46.3)	0.001	28.7 (26.1–31.4)	0.003	29.3 (26.4–32.2)	0.001	15.5 (14.0–17.1)	0.90	16.0 (14.4–17.6)	0.58
	Absolute**	2432 (2072–2792)	0.02	2395 (2009–2781)	0.022	1479 (1255–1703)	0.79	1510 (1272–1747)	0.49	849 (713–985)	0.26	868 (721–1015)	0.94
**50–1000**	**Relative**	48.7 (46.2–51.3)	0.74	48.6 (46.1–51.0)	0.99	23.8 (21.3–26.4)	0.65	24.2 (21.7–26.7)	0.96	15.8 (14.4–17.2)	0.68	15.6 (14.2–17.0)	0.73
	**Absolute**	2872 (2533–3210)	0.94	2888 (2548–3229)	0.96	1375 (1165–1585)	0.53	1416 (1206–1625)	0.57	952 (824–1079)	0.77	947 (817–1077)	0.80
**<50**	**Relative**	48.3 (47.3–49.2)	Ref	48.5 (47.6–49.5)	Ref	24.5 (23.5–25.4)	Ref	24.1 (23.2–25.1)	Ref	15.4 (14.9–16.0)	Ref	15.5 (15.0–16.1)	Ref
	**Absolute**	2884 (2759–3010)		2878 (2751–3005)		1446 (1369–1523)		1419 (1342–1497)		931 (884–979)		930 (882–979)	

*Relative counts are reported as percentages.

**Absolute counts are reported as cells/mm^3^.

^†^Comparing means in group VL > 1000 and VL 50–1000 to VL < 50 (reference group) using sum of least squares.

^‡^Adjusted for maternal CD4, maternal ART use, infant ART use, gender, gestational age and race in a multivariable analysis of variance.

**Table 3:** Infant CD4, CD19 and CD8 parameters at 6 months of age

**Maternal viral load**		**CD4 (mean, 95% CI)**	**p**^**†**^	**Adjusted CD4 (mean, 95% CI)**	**p**^**‡**^	**CD19 (mean, 95% CI)**	**p**^**†**^	**Adjusted CD19 (mean, 95% CI)**	**p**^**‡**^	**CD8 (mean, 95% CI)**	**p**^**†**^	**Adjusted CD8 (mean, 95% CI)**	**P**^**‡**^
>1000	Relative*	42.1 (39.3–44.9)	0.009	40.1 (37.1–43.1)	<0.001	30.8 (27.9–33.7)	0.008	32.9 (29.8–36.1)	<0.001	15.2 (13.6–16.8)	0.99	14.9 (13.2–16.6)	0.73
	Absolute**	2271 (1907–2635)	0.002	2290 (1893–2687)	0.022	1528 (1261–1794)	0.27	1712 (1420–2003)	0.73	828 (680–977	0.10	853 (690–1016)	0.95
50-1000	Relative	47.0 (44.5–49.4)	0.52	46.8 (44.3–49.2)	0.78	27.0 (24.5–29.6)	0.75	27.8 (25.2–30.4)	0.27	15.8 (14.4–17.1)	0.43	15.3 (13.9–16.7)	0.24
	Absolute	2861 (2540–3181)	0.88	2876 (2550–3202)	0.96	1727 (1492–1961)	0.76	1788 (1549–2027)	0.32	937 (806–1068)	0.74	926 (792–1060)	0.68
<50	Relative	46.1 (45.1 – 47.1)	Ref	46.4 (45.4–47.4)	Ref	26.6 (25.5–27.6)	Ref	26.2 (25.2–27.3)	Ref	15.2 (14.6–15.7)	Ref	15.3 (14.7–15.8)	Ref
	Absolute	2887 (2756–3017)		2878 (2747–3010)		1687 (1592–1783)		1656 (1559–1752)		961 (908–1014)		957 (903–1011)	

*Relative counts are reported as percentages.

**Absolute counts are reported as cells/mm^3^.

^†^Comparing means in group VL > 1000 and VL 50–1000 to VL < 50 (reference group) using sum of least squares.

^‡^Adjusted for maternal CD4, maternal ART use, infant ART use, gender, gestational age and race in a multivariable analysis of variance.

#### Infant CD19^+^ B cell counts

There was a significant difference in the percentage of CD19^+^ B cells among infants born to mothers with HIV-1 VL > 1,000 *vs.* < 50 RNA copies/ml plasma at 2 and 6 months of age, with higher CD19^+^ B cell percentages among infants born to mothers with higher VL (>1,000 copies) compared to those whose mothers were undetectable at the time of delivery (28.7% *vs.* 24.5%, p = 0.003, and 30.8% *vs.* 26.6%, p =0.008, respectively).

This difference remained significant after adjusting for gestational age, race, gender, maternal and infant ART exposure, and maternal CD4^+^ T cell counts at the time of delivery (p = 0.01 and p < 0.001, respectively). The differences in absolute CD19 count at 2 and 6 months of age (1479 vs. 1446 cells/mm^3^ and 1528 vs. 1687 cells/mm^3^) were not statistically significant (p = 0.79 and p = 0.27, respectively), both in the unadjusted and adjusted analysis.

#### Infant CD8^+^ T cell counts

There was no significant difference in CD8^+^ T cell count (absolute or percentage) among infants born to mothers with HIV-1 VL > 1000 *vs.* < 50 RNA copies/ml plasma at 2 months of age (15.5% *vs.* 15.4%, p = 0.90 and 849 vs. 931, p = 0.26) nor at 6 months of age (15.2 vs. 15.2, p = 0.99 and 828 vs 961 cells/mm^3^, p = 0.10), both unadjusted and adjusted.

## Discussion

This study of HEU infants in Canada revealed differences in infant CD4^+^ T cell counts as a function of maternal HIV-1 VL measured at the time of delivery. Decreased CD4^+^ T counts (relative and absolute) were observed at 2 and 6 months of age in infants born to mothers with a VL > 1000 copies/ml at the time of delivery, when compared to infants born to mothers with undetectable viral load (<50 copies/ml). Our findings are consistent with a recent study from Malawi [40] which also found decreased CD4% at one month of age among HEU infants compared to unexposed uninfected controls, with CD4 values (mean 44% IQR 39–50) similar to those reported in the present study among 2 month old infants born to mothers with VL > 1000 copies/ml (mean 44.4, 95% CI 41.3-47.5). Though the specific mechanisms behind this association have yet to be understood, it has been shown that HIV-infected, ART-treated women have greatly increased placental production of inflammatory cytokines, tumor necrosis factor alpha (TNF-α), and interleukin 8 (IL-8) compared to uninfected women [41,42], and mothers with high VL have been shown to generate higher levels of the pro-inflammatory cytokines INF-δ and TNF-α [43]. This pro-inflammatory, immune activated intra-uterine state is likely be increased in women with poorly controlled HIV-1 viremia, and could potentially be the driving force behind the differences in CD4 count seen in this study among the infants born to mothers defined by extremes of maternal viral control (<50 vs. >1000 copies/ml).

The significance of the changes in CD19+ B cell counts is not clear. Infants born to mothers with VL > 1000 copies/ml at the time of delivery had an increased CD19^+^ B cell percentage, though no change was seen in their absolute CD19+ B cell count. To date, B cell function among HEU infants is not well understood. Some studies have shown a more robust antibody response to vaccines among HEU infants as compared to unexposed, uninfected controls [44,45]. When compared to Hepatitis C exposed infants, HEU infants have been shown to have lower levels of transferred maternal antibodies at birth and significantly higher total immunoglobulin levels until 2 years of age [46], and it is thought that reduced maternal antibody interference with vaccines may contribute to a stronger vaccine response. However, others studies have reported decreased neutralizing-antibody titres to the oral poliovirus vaccine [47] and diminished response to the Hepatitis B vaccine among HEU infants [48]. Given the uncertainty surrounding B cell function among HEU, it is not clear if our findings of decreased CD19% in the absence of changes to absolute CD19 count are suggestive of an altered overall immunological profile in response to aggravated HIV exposure, or simply represent an isolated finding.

Our study is limited by our ability to control for other confounders that are both known and hypothesized to affect infant immune status, including malnutrition, recreational exposure to hard drugs, and cytomegalovirus co-infection status [21]. While all women in our cohort received identical treatment by the same team of caregivers under a system of universal healthcare access (including social welfare benefits), we were unable to adjust for individual differences in overall health, nutritional, socioeconomic and social status. Most importantly, the clinical implications of these subtle differences in CD4^+^ T cell counts among HEU infants are not clear. Extrapolating from studies in endemic settings, they may translate into an increased susceptibility to infectious diseases. HEU infants born to severely immunocompromised mothers (CD4 < 200 cells/mm^3^) in a South African cohort were found to be at higher risk of developing early- and late-onset sepsis than those born to mothers with CD4 > 350 cells/mm^3^[49]. Data from the Ban trial in Malawi further demonstrated that low total white blood cell counts at birth are a significant predictor of serious morbidity and mortality among HEU infants [50]. The small differences in CD4 count seen in the present study may further be compounded by functional immune abnormalities, something that was not specifically examined.

## Conclusion

In conclusion, the results of this study may contribute to an understanding of the immune system of HEU infants, and provide insight into the causes of the increased morbidity and mortality from infectious diseases seen among HEU infants in HIV-endemic settings. Mothers with higher levels of HIV-1 viremia are likely to be sicker, experience faster disease progression, and be less able to care for their infants. These infants, though uninfected, possibly take 2 hits: a relative state of immunodeficiency marked by decreased CD4^+^ T cell count and decreased placental transfer of protective maternal antibodies, and increased exposure to infectious diseases from sicker parents. While in well-resourced settings this may have little clinical impact given access to treatment and care, in resource-limited settings, susceptibility to even mild, self-limited diseases such as upper respiratory tract infections or gastroenteritis could lead to significant infant morbidity and mortality. In short, these results reinforce the need for optimal treatment of all HIV-infected pregnant women, both to prevent perinatal HIV transmission, and to limit possible adverse events resulting from *in utero* exposure to high level HIV viremia. Further studies are needed to detail the immunological response among these infants, and to determine any functional immune abnormalities.

## Abbreviations

3TC: Lamivudine; ART: Antiretroviral therapy; AZT: Zidovudine; CHU: Centre hospitalier universitaire; CMIS: Centre maternel et infantile sur le SIDA; HEU: HIV-exposed uninfected; HIV-1: Human immunodeficiency virus type 1; PMTCT: Prevention of mother to child transmission.

## Competing interests

The authors declare that they have no competing interests.

## Authors’ contributions

FK conceptualized the study, participated in the statistical analysis, and drafted the final manuscript. VL contributed to the study design and writing of the final manuscript. TD conducted the statistical analysis. SV collected and did a preliminary analysis of the data. HS participated in the design of the study, interpretation of findings, and helped draft the final manuscript. NL established and was responsible for the management of the cohort, conducted the immunological analysis, and contributed to the final manuscript. All authors read and approved the final manuscript.

## Pre-publication history

The pre-publication history for this paper can be accessed here:

http://www.biomedcentral.com/1471-2334/14/236/prepub

## Supplementary Material

Additional file 1: Table S1 Multiple linear regression models. Click here for file
