# Supplementary material for: Impact of maternal HIV-1 viremia on lymphocyte subsets among HIV-exposed uninfected infants: protective mechanism or immunodeficiency
Source: BMC Infect Dis. 2014 May 5;14:236. doi: 10.1186/1471-2334-14-236 (PMC4024098; doi:10.1186/1471-2334-14-236)
Supplement: Additional file 1: Table S1 — Multiple linear regression models. [file 1471-2334-14-236-S1.docx]

**Supplementary Table: Multiple linear regression Models**

| Model | 2 month CD4 absolute | 2 month CD4 percentage | 2 month CD19 absolute | 2 month CD19 percentage | 6 month CD4 absolute | 6 month CD4 percentage | 6 month CD19 absolute | 6 month CD19 percentage |
| --- | --- | --- | --- | --- | --- | --- | --- | --- |
| **R2** | 0.079 | 0.141 | 0.077 | 0.093 | 0.082 | 0.123 | 0.056 | 0.075 |
| **Variable** | ***p value*** | ***p value*** | ***p value*** | ***p value*** | ***p value*** | ***p value*** | ***p value*** | ***p value*** |
| Infant ARV | **0.057** | **0.005** | 0.134 | **0.023** | 0.336 | **0.013** | 0.876 | 0.600 |
| Maternal  ARV | 0.761 | 0.282 | 0.782 | 0.357 | 0.404 | 0.420 | 0.551 | 0.454 |
| Viral Load | 0.063 | 0.017 | 0.860 | 0.008 | 0.011 | 0.016 | 0.534 | 0.015 |
| CD4 | 0.237 | 0.720 | 0.433 | 0.889 | 0.122 | 0.870 | 0.081 | 0.410 |
| Gender | 0.001 | <0.001 | 0.301 | 0.017 | 0.011 | 0.006 | 0.8071 | 0.345 |
| Race | 0.657 | 0.018 | <0.001 | 0.006 | 0.082 | 0.005 | 0.003 | 0.002 |
